# Platelet lysate can support the development of a 3D-engineered skin for clinical application

**DOI:** 10.1007/s00441-022-03698-7

**Published:** 2022-10-22

**Authors:** I. Banakh, Md. M. Rahman, C. L. Arellano, D. C. Marks, S. Mukherjee, C. E. Gargett, H. Cleland, S. Akbarzadeh

**Affiliations:** 1 Skin Bioengineering Laboratory, Victorian Adult Burns Service, Alfred Health, 89 Commercial Road, Melbourne, Vic 3181 Australia; 2 Department of Surgery, Monash University, 99 Commercial Road, Melbourne, Vic Australia; 3 Australian Red Cross Lifeblood, 17 O’Riordan Street, Alexandria, NSW Australia; 4 Sydney Medical School, University of Sydney, Camperdown, NSW Australia; 5 The Ritchie Centre, Hudson Institute of Medical Research, Clayton, Vic Australia

**Keywords:** Platelet lysate, Primary keratinocytes culture, Senescence, TGF-β1, Collagen IV

## Abstract

Safety concerns associated with foetal bovine serum (FBS) have restricted its translation into clinics. We hypothesised that platelet lysate (PL) can be utilised as a safe alternative to produce serum-free 3D-engineered skin. PL supported a short-term expansion of fibroblasts, with negligible replication-induced senescence and directed epidermal stratification. PL-expanded fibroblasts were phenotypically separated into three subpopulations of CD90^+^FAP^+^, CD90^+^FAP^−^ and CD90^−^FAP^+^, based on CD90 (reticular marker) and FAP (papillary marker) expression profile. PL drove the expansion of the intermediate CD90^+^ FAP^+^ subpopulation in expense of reticular CD90^+^FAP^−^, which may be less fibrotic once grafted. The 3D-engineered skin cultured in PL was analysed by immunofluorescence using specific markers. Detection of ColIV and LMN-511 confirmed basement membrane. K10 confirmed near native differentiation pattern of neo-epidermis. CD29- and K5-positive interfollicular stem cells were also sustained. Transmission and scanning electron microscopies detailed the ultrastructure of the neo-dermis and neo-epidermis. To elucidate the underlying mechanism of the effect of PL on skin maturation, growth factor contents in PL were measured, and TGF-β1 was identified as one of the most abundant. TGF-β1 neutralising antibody reduced the number of Ki67-positive proliferative cells, suggesting TGF-β1 plays a role in skin maturation. Moreover, the 3D-engineered skin was exposed to lucifer yellow on days 1, 3 and 5. Penetration of lucifer yellow into the skin was used as a semi-quantitative measure of improved barrier function over time. Our findings support the concept of PL as a safe and effective serum alternative for bioengineering skin for cell therapies.

**Supplementary Information:**

The online version contains supplementary material available at 10.1007/s00441-022-03698-7.

## Introduction

In recent years, allogeneic platelet-derived biomaterial has attracted attention for the treatment of difficult-to-heal wounds due to its antimicrobial activity and high content of growth factors (Akbarzadeh et al. 2021; Shariati et al. 2020). In vitro, in the absence of fully defined media for the expansion of a variety of cell types, platelet lysate (PL) has been used as a source of stimulatory growth factors and an effective substitute for FBS during manufacturing steps in a variety of cell therapies, particularly mesenchymal stromal cells (MSCs) (Haack-Sorensen et al. 2018). Replacing FBS with PL as a cell culture supplement not only improves the regulatory concerns to the clinical product by reducing a risk of prions and virus transmission, but also has ethical and animal welfare benefits by eliminating the requirement for bovine foetuses during the process. Furthermore, PL is often prepared from outdated platelets collected for clinical applications, which would otherwise be disposed of, and therefore reduces wastage (Astori et al. 2016; Bieback et al. 2019).

Few studies have investigated the efficacy of platelet-derived biomaterial for primary dermal and epidermal cell expansion as therapeutics. One per cent and 5% platelet-rich plasma (PRP) has been shown to increase ColI synthesis and upregulate β1-integrin receptor, focal adhesion kinase and phosphorylated mitogen-activated protein kinases in skin fibroblasts (Guszczyn et al. 2017). Moreover, PL at a higher concentration (20%) promoted fibroblast colIII matrix deposition (Ranzato et al. 2011). Whether such stimulatory effects of platelet-derived biomaterial would contribute to faster wound healing in vivo has not been tested.

PL at 2.5% and 5% has been reported to replace FBS for short-term keratinocyte viability; however, the long-term effect of PL on keratinocyte stratification and differentiation has not been fully explored (Altran et al. 2013). It also has been shown to enhance cell proliferation and migration in epidermal cell lines and primary keratinocytes (Barsotti et al. 2013; El Backly et al. 2011; Misiura et al. 2021). This was mediated through actin cytoskeletal re-organisation that persisted for up to 24 h. The PL-enhanced keratinocyte migration was associated with a high expression of the inflammatory cytokine interleukin-8 and the activation of p38 mitogen-activated protein kinase and NF-кB pathways (Barsotti et al. 2013; El Backly et al. 2011). Moreover, the expression of keratinocyte differentiation markers, involucrin and transglutaminase-1, was reported in primary keratinocytes expanded at higher PL concentrations (10%) (Bayer et al. 2017).

Previously, we have shown that human-derived serum allows animal-free expansion of adult fibroblasts and keratinocytes as monolayers (Cheshire et al. 2019). Here, we hypothesise that PL can replace serum (either animal- or human-derived) in culture, if fibroblasts and keratinocytes are co-cultured and allowed to mature in a 3D-engineered skin. This will allow skin maturation that resembles native skin architecture. Previously, we have developed a platelet-derived hydrogel as a scaffold to support epidermal stratification and maturation in a 3D-engineered skin (Rahman et al. 2021). In this study, we investigated whether PL, as a media supplement, can support neo-epidermal formation in a 3D model by analysing keratinocyte proliferation, stratification, differentiation and barrier function. The effect of PL on neo-epidermal formation was also measured indirectly through measuring fibroblast proliferation, senescence, growth factor expression and extracellular matrix (ECM) deposition in presence of PL. PL-expanded fibroblasts were divided into CD90^+^FAP^+^, CD90^+^FAP^−^ and CD90^−^FAP^+^ subpopulations using FAP (fibroblast-activation protein) and CD90 (cluster of differentiation 90 or Thy-1) cell surface markers (Korosec et al. 2019). The effects of PL on expansion of CD90^+^FAP^−^ (reticular phenotype), CD90^+^FAP^+^ (intermediate phenotype) and CD90^−^FAP^+^ (papillary phenotype) fibroblasts subpopulations were determined.

## Methods

### Access to human-derived material

This project was approved by the Australian Red Cross Lifeblood (Lifeblood) (Approval Number C Loh 18,072,014) and Alfred Health Ethics Committee (Approval Number 269/17). Skin tissue discarded during elective breast reduction or abdominoplasty surgery was obtained after informed consent. This work was carried out in accordance with The Code of Ethics of the World Medical Association (Declaration of Helsinki) for experiments involving humans.

### Platelet-derived lysate and hydrogel preparation

Platelet concentrates that were negative for bacterial contamination were frozen at − 80 °C within one day after expiry. PL batches were produced from platelet concentrates (Australian Red Cross Lifeblood) of either a single blood group or a mixed blood group. Platelets were thawed at 2–6 °C for 24 h, and centrifugation was used to remove debris/precipitate (5005 g, 10 min). Freezing, thawing and centrifugation processes were repeated. Batches of PL were pooled from 10–12 individual concentrates. These were then subdivided and frozen at − 80 °C.

Platelet-derived hydrogel was produced as described previously (Rahman et al. 2021). Total protein concentration was measured using a bicinchoninic assay (BCA) according to the manufacturer’s instructions (Thermo Fisher Scientific, Waltham, MA, USA). Growth factor concentrations were analysed using Quantikine ELISA kits (R&D Systems, Minneapolis, MN, USA) according to the manufacturer’s instructions.

### Isolation and expansion of adult keratinocytes and dermal fibroblasts

Keratinocytes were isolated and cultured as previously described (Banakh et al. 2020). Dermal fibroblasts were isolated and cultured as described by Boyce (1999) with minor modifications. Isolated cells were seeded and expanded in low glucose DMEM with hydrocortisone (0.5 µg/ml, Merck, Billerica, USA), insulin (50 IU/ml, Novo Nordisk, Australia), gentamicin (50 µg/mL, Life Technologies, Carlsbad, USA) and FBS (4%, Cytiva, Marlborough, USA) or PL (0.5%, 1%, 2% or 4%).

### 3D serum-free engineered skin

Dermal fibroblasts were seeded either within, at the time of gelation, or on top of the hydrogel, after gelation. Keratinocytes were seeded on top of the hydrogel and 3D skin was cultivated in UCDM1 medium supplemented with PL for 8 days (Boyce 1999). Where noted, transforming growth factor β1 (TGF-β1) neutralising antibody (Abcam, Waltham, USA) or IgG1 (DAKO, Glostrup, Denmark) was added to media (1:10,000) during the cultivation period.

### IL-6 and IL-8 ELISA

Human fibroblasts were cultured in 6-well plates for 3 weeks with 4% FBS, 2% PL and 4% PL-supplemented medium. Supernatants were assessed for human IL-6 and IL-8 content using ELISA (BD Bioscience, San Diego, USA) according to the manufacturer’s instructions.

### Transmission electron microscopy (TEM)

Tissue samples were cut into cubes of 1 mm^3^ and placed into primary fixative, consisting of 2.5% glutaraldehyde and 2% paraformaldehyde in 0.1 M sodium cacodylate buffer, for 1 h at room temperature followed by overnight incubation at 4 °C. The tissues were then rinsed in fresh 0.1 M sodium cacodylate buffer three times for 15 min each. Secondary fixation was performed using 1% osmium tetroxide and 1.5% potassium ferricyanide in 0.1 M cacodylate buffer for 1 h at RT. The tissues were rinsed in three washes of milli-Q water for 15 min each. The fixed tissues were dehydrated by incubating in increasing concentrations of ethanol for 15 min, consisting of 30, 50, 70, 90 and 100% ethanol. The ethanol was removed and replaced with 100% propylene oxide. Dehydrated tissues were incubated in a mixture of Epon resin and propylene oxide at a ratio of 1:1 for 6 h at room temperature, followed by a 2:1 Epon/propylene oxide mixture overnight. Tissues were incubated in 100% freshly made Epon resin for 6 h, followed by another 100% resin change overnight. The tissues were then placed into Beem capsules in 100% resin, and the resin was polymerised for 48 h in an oven at 60 °C. Resin-embedded tissue was sectioned with a Diatome diamond knife using a Leica UCS ultramicrotome. Sections of thickness 70–90 nm were collected onto 150 mesh copper/palladium grids and stained sequentially with 1% uranyl acetate for 5 min and lead citrate for 5 min. The sections were imaged in a JEOL 1400 + transmission electron microscope at 80 kV, and images were captured with a digital camera at a resolution of 2 K × 2 K.

### Scanning electron microscopy (SEM)

All biological samples were fixed with 4% PFA frozen in OCT at − 80°C. The sections were dehydrated with ascending concentrations of ethanol (50%, 75%, 95%, 100%) for 15 min each. All samples were treated with hexamethyldisilazane (HMDS), air-dried overnight and mounted on a conductive aluminium surface using carbon tapes. Samples were coated with a thin gold layer (Bal-Tec SCD 005, Leica USA) prior to examination under the scanning electron microscope (Nova Nano- SEM, FEI, USA). Images were acquired at 5 mm working distance under a beam of 10 kV and quantified using Image J software (NIH).

### Proliferation assay

Dermal fibroblast proliferation was measured using AlamarBlue (Bio-Rad Laboratories Inc, Hercules, USA) according to the manufacturer’s instructions. Briefly, triplicate seedings of cells were left to attach in a 24-well plate. Media was replaced with 10% Alamar Blue stock in media and incubated at 37 °C for 2 h. Finally, 100 µl aliquots from each well were transferred to a 96-well plate. Fluorescence measurements at 590 nm emission were collected using a Fluostar Optima plate reader (BMG Labtech, Ortenberg, Germany). Long-term fibroblast proliferation was monitored by cell counting, using trypan blue exclusion, when 70–80% confluence was reached at each split.

### Absolute telomere length measurement

Dermal fibroblast’s DNA was extracted using QIAmp (Qiagen, Hilden, Germany) kit and quantitated on Quantus Fluorometer using QuantiFluor ONE dsDNA kit. QPCRs were performed as described previously (O'Callaghan and Fenech 2011). Briefly, 20 ng DNA per well was loaded in triplicate in 96-well plates with GoTaq qPCR Master mix (Promega), 5′-cggtttgtttgggtttgggtttgggtttgggtttgggtt-3′ forward and 5′-ggcttgccttacccttacccttacccttacccttaccct-3′ reverse primers. Standard curves were generated from the dilution of known quantities of telomere standard, 5′-(TTAGGG)_14_–3′. Standard DNA content was topped to 20 ng with pBR322 plasmid (Life Technologies).

### β-galactosidase assay

Senescence-associated β-galactosidase was detected as described (Dimri et al. 1995). Briefly, 30 × 10^3^ fibroblasts were seeded onto glass coverslips. Cells were fixed in 3% formaldehyde (3 min) and stained in fresh β-Galactosidase staining solution at 37 °C, overnight. Stained coverslips were washed in PBS before light microscopy analysis.

### Growth factor and ECM expression

RNA was extracted using Trizol as previously described (Banakh et al. 2020). cDNA was prepared from 400 ng of RNA using a GoScript Reverse Transcriptase Kit (Promega, Madison, USA). qPCR was performed in a LightCycler® 480 Multiwell Plate 384 (Roche, Basel, Switzerland) using Go Taq qPCR Master Mix (Promega, Madison, USA) according to the manufacturer’s instructions. Primers used to detect growth factors, ECM markers and housekeeping genes in fibroblasts were as follows: KGF (5′-ttgtggcaatcaaaggggtg-3′, 5′-cctccgttgtgtgtccatttagc-3′) (Narita et al. 2009), FGF-2 (5′-gaagagcgaccctcacatcaagcta-3′, 5′-cagttcgtttcagtgccacatacc-3′) (Narita et al. 2009), IL-6 (5′-ggtacatcctcgacggcatct-3′, 5′-gtgcctctttgctgctttcac-3′) (Keller et al. 2003), IL-8 (5′-actgagagtgattgagagtggac-3′, 5′-aaccctctgcacccagttttc-3′) (Tsai et al. 2009), Col1A1 (5′-atgtctagggtctagacatgttca-3′, 5′-ccttgccgttgtcgcaga-3′) (Tancred et al. 2009), Col3A1 (5′-agctgttgaaggaggatgttcc-3′, 5′- ctgcgagtcctcctactgct-3′), FN1 (5′-caactcactgacctaagctttgt-3′, 5′-ggtgaatcgcaggtcagt-3′), GAPDH (5′-ctctgctcctcctgttcgac-3′, 5′-aaatgagccccagccttctc-3′), HPRT (5′-attggtaatgaccagtaccagtcaacag-3′, 5′-gcattgttttgccagtgtcaa-3′) and TBP (5′-cacgaaccacggcactgatt-3′, 5′-ttttcttgctgccagtctggac-3′) (Moore et al. 1999).

### Flow cytometry

Dermal fibroblasts cultured in 4% FCS, 2% PL or 4% PL supplemented media were mixed with Efluor 450 viability dye (Thermo Fisher, Eugene, USA) for 30 min at 4°C. Cell aliquots were washed in PBS after fixation, labelling and permeabilization steps. Cells were fixed in 1% formaldehyde and blocked in 2% bovine serum albumin (MP Biomedicals, Auckland, New Zealand)/2% FBS, 15 min at 4°C. Cells were stained with CD90-BV605 antibody (BioLegend, San Diego, USA, 1:10) and FAP-APC (R&D Systems, Minneapolis, USA, 1:10) for 30 min at 4°C under agitation. Samples were permeabilised in 0.2% Triton-X (Sigma-Aldrich). Cells were then incubated with mouse anti-vimentin (DAKO, 1:100), rabbit anti-podoplanin (Abcam, Cambridge, USA, 1:50) and mouse anti-transglutaminase 2 (Abcam, 1:100) antibodies, followed by incubation with anti-mouse Alexa Fluor 488 (Invitrogen, Waltham, USA, 1:400) and anti-rabbit Ig-PE (Invitrogen, 1:400). Analysis was carried out using Fortessa (BD Biosciences) and Flowlogic software (Inivai Technologies, Mentone, Australia).

### Immunofluorescence

Cryopreserved sections were blocked with 5% BSA/5% horse serum (Sigma) and incubated overnight with primary antibodies: rabbit anti-human K5 (1:500, Biolegend), mouse anti-human K10 (1:500, DAKO), mouse anti-human Col IV (1:500, Sigma), rabbit anti-human CD29 (1:200, GeneTex, Irvine, USA), mouse anti-human Ki67 (1:100, DAKO), mouse anti-human Vimentin (1:200, DAKO), rabbit anti-human pan cytokeratin (1:100, Novus Biological, Littleton, USA) or mouse anti-human α5 chain Laminin (1:500, Millipore, Burlington, USA). Slides were washed and incubated with Alexa Fluor-conjugated mouse or rabbit secondary antibodies (BD Biosciences, Columbus, USA). Sections were imaged on a Nikon Ti-E microscope. Ki67 staining was quantitated for fluorescent nuclei with the ‘Tissue Analysis Cell Counter’ macro using FIJI software (NIH). Ki67 and DAPI nuclear signal thresholds were adjusted to minimise nuclear segmentation or fusion and verified by manual count.

### Lucifer yellow barrier function

Fresh tissue was incubated with lucifer yellow (1 mg/mL) for 10 min at room temperature on days 1, 3 and 5. Excess dye was blotted and tissues were fixed in PFA. Sections were stained for laminin-511 and analysed by confocal microscopy.

## Results

### PL can support adult dermal fibroblast short-term expansion

**Fig. 1:**
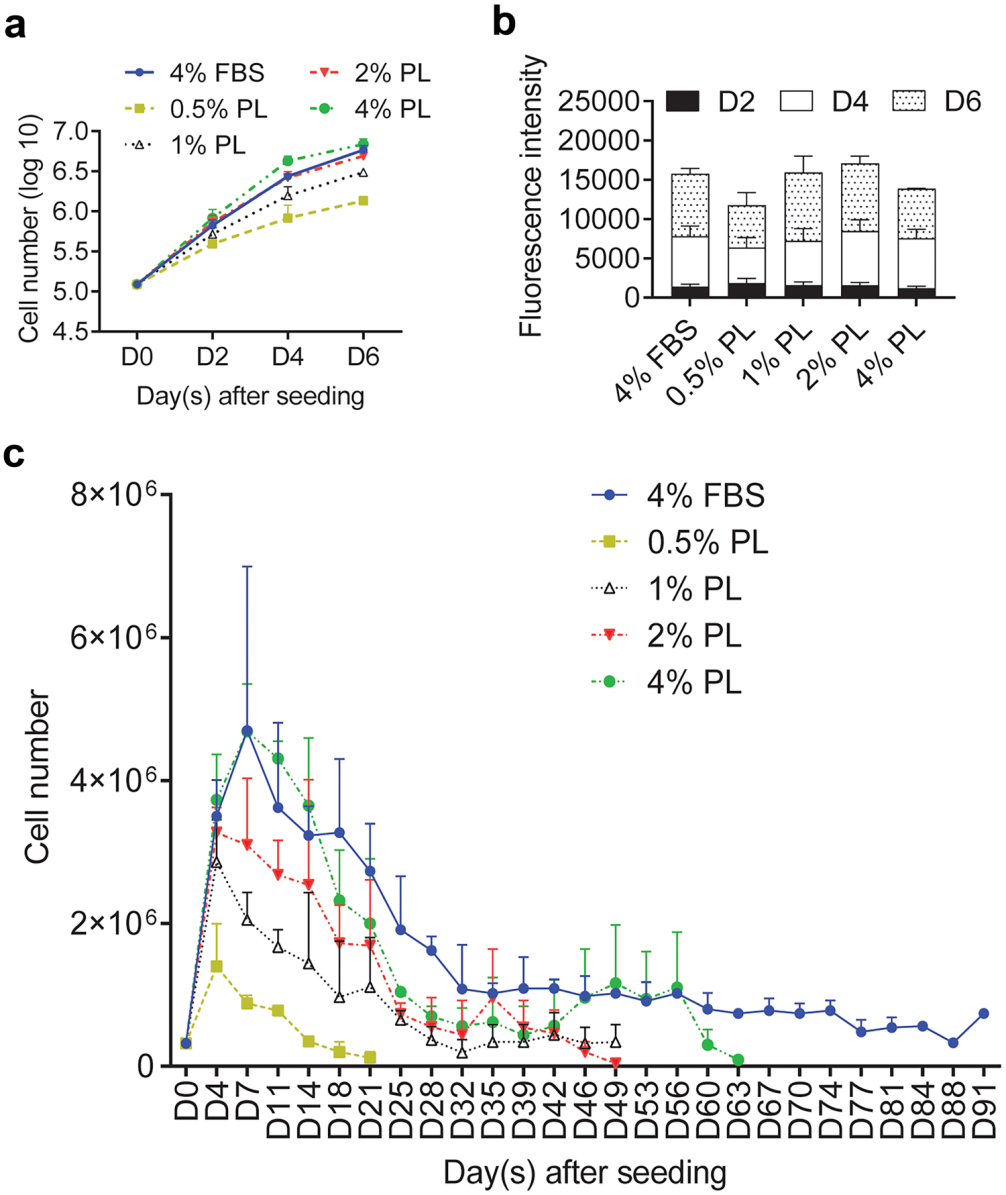
Platelet lysate (PL) can support expansion of dermal fibroblasts ex vivo. Primary adult fibroblast proliferation in media supplemented with 0.5%, 1%, 2% and 4% PL, compared to 4% FBS were measured by **a** cell counting and **b** Alamar Blue on day 2, day 4 and day 6 post-seeding. **c** Fibroblasts were kept in media supplemented with 0.5% PL, 1% PL, 2% PL, 4% PL or 4% FBS for over 3 months. Cell numbers were determined at each split. Values represent mean + / − SEM in each group (*n* = 3 per group). Data analysed using an unpaired *t*-test

To avoid batch-to-batch variation, PL was prepared by pooling lysates from 10–12 individuals. Three batches were tested. Fibroblasts were isolated and cultured in media supplemented with 0.5%, 1%, 2% and 4% PL and compared to 4% FBS in both short-term (6 days) and long-term (over 90 days) cultures. PL (2% and 4%) supported fibroblast expansion in a similar manner to 4% FBS over a 6-day period (Fig. 1a and b). A slight decline in the growth rate of PL-supplemented long-term cultures was not significant (Fig. 1c). However, fibroblast population growth rate decline in 2% and 4% PL cultures led to a drop below the required expansion numbers after 49 and 63 days, respectively. Fibroblasts cultured in 4% FBS continued to grow over 90 days. Moreover, there were no obvious morphological differences between dermal fibroblasts grown in 4% FBS control and 2% PL, whereas long-term culture in 4% PL caused stress, evident from increased cell membrane filopodia (data not shown). Subsequently, 2% PL was used to supplement media for short-term, animal-free, serum-free fibroblast expansion.

### Long-term fibroblast expansion in PL triggers senescence

**Fig. 2:**
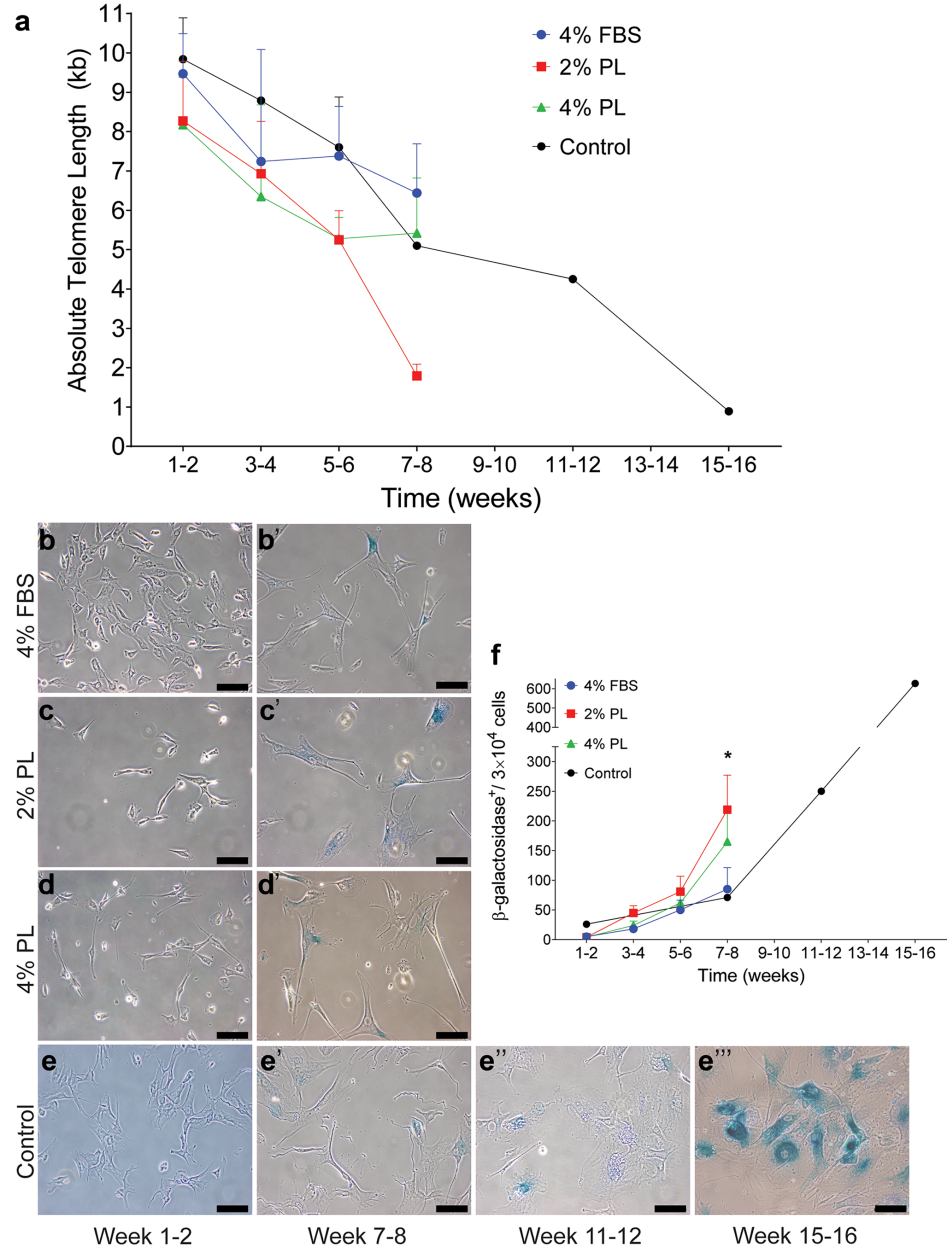
Replication-induced senescence in fibroblasts expanded in PL-supplemented media. Senescence in fibroblasts expanded in 2% and 4% PL, compared to 4% FBS, was quantified by measuring **a** absolute telomere length and **b**–**f** β-galactosidase accumulation in cells. Panels **b**–**e** and **b’**–**e’** visualise, weeks 1–2 and weeks 7–8 stained fibroblast cultures, respectively. Panels **e’’** and **e’’’** present stained fibroblasts from weeks 11 to 12 and weeks 15 to 16 cultures, respectively. Fibroblasts in 4% FBS-supplemented standard media were kept in culture for up to 16 weeks as a reference. Values represent mean values + / − SEM in each group (* = *p* ≤ 0.05, ** = *p* ≤ 0.01, *** = *p* ≤ 0001, *n* = 5 per group)

Replication-induced senescence in long-term expanded fibroblasts was measured by two independent methods. Firstly, absolute telomere length was calculated by qPCR in fibroblast expanded in 2% PL and 4% PL compared to 4% FBS supplemented medium (Fig. 2a). As expected, there was a slow decline in absolute telomere length over time, regardless of the culture supplement. Secondly, β-galactosidase accumulation was measured using β-gal staining (Fig. 2b–e’’’). Fibroblasts cultured in 2% PL showed increased accumulation of β-galactosidase after 7–8 weeks of expansion (Fig. 2f, p = 0.0201). No accumulation was observed at any earlier time point. Overall, these data suggest that replication-induced senescence may be induced when fibroblasts are cultured in PL over a longer period of time. However, there is no evidence that shorter PL expansion of fibroblasts (i.e. up to 6 weeks) triggers senescence.

### PL affects fibroblast and keratinocyte expansion through its rich growth factor content

**Fig. 3:**
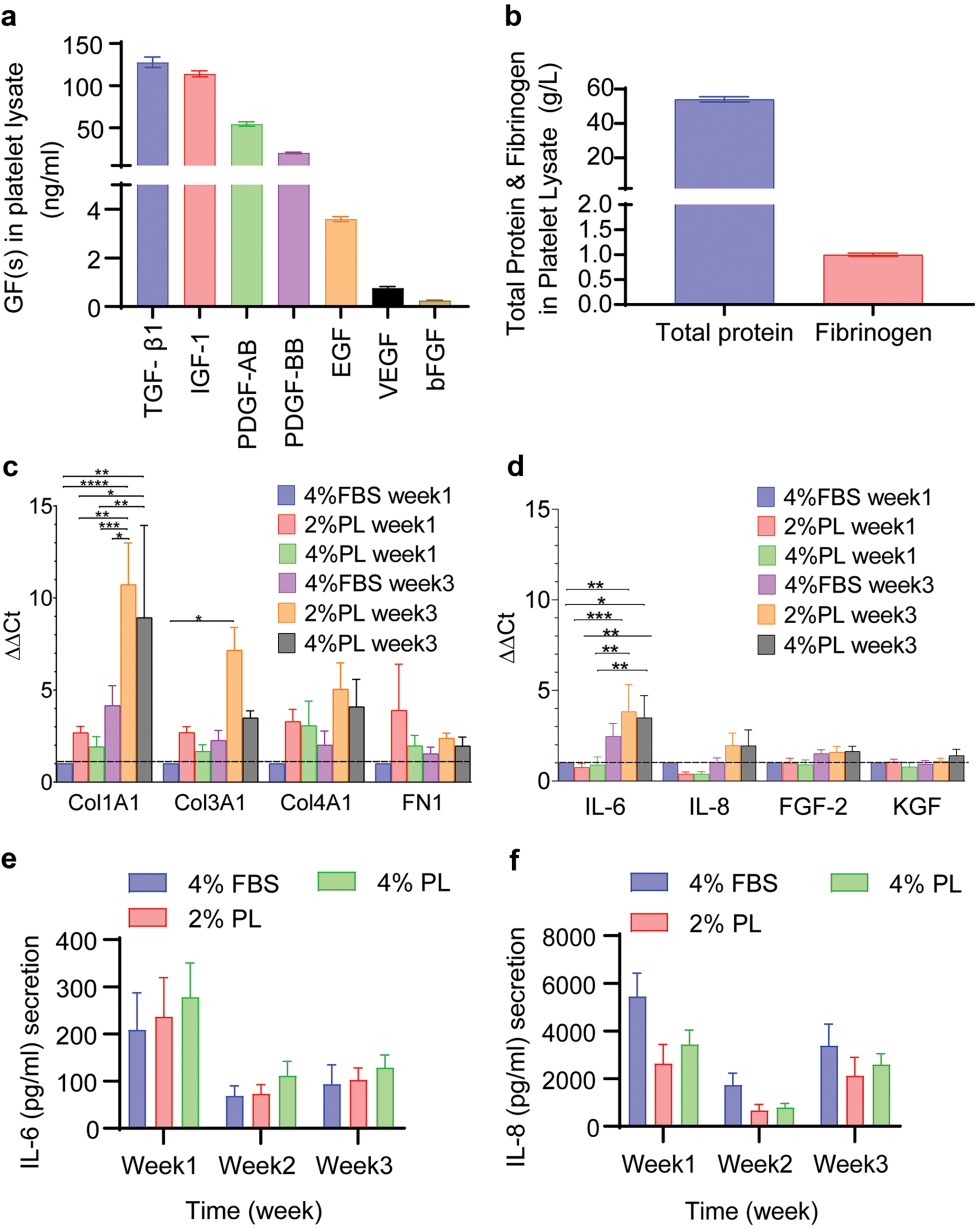
PL contains a number of growth factors that stimulate fibroblasts. **a** transforming growth factor β1 (TGF-β1), insulin-like growth factor 1 (IGF-1), platelet-derived growth factors (PDGF-AB, and PDGF-BB), epidermal growth factor (EGF), vascular endothelial growth factor (VEGF) and basic fibroblast growth factor (bFGF or FGF-2) concentrations in PL (*n* = 8). **b** Total protein and fibrinogen concentrations in PL (*n* = 8) **c**,**d** collagen 1a1 (Col1A1), collagen 3a1 (Col3A1), collagen 4a1 (Col4A1) and fibronectin-1 (FN1), Interleukin-6 (IL-6), Interleukin-8 (IL-8), fibroblast growth factor-2 (FGF-2) and keratinocyte growth factor (KGF) expression were analysed in fibroblasts expanded in 2% and 4% PL over 3 weeks, compared to 4% FBS. Graphs represent mean values + / − SEM in each group (*n* = 5 per group) (* = *p* ≤ 0.05, ** = *p* ≤ 0.01, *** = *p* ≤ 0.001). **e**–**f** IL-6 and IL-8 secretion by fibroblasts isolated and expanded in 2% PL-, 4% PL- or 4% FBS-supplemented media was measured by ELISA after 1, 2 and 3 weeks culture (*n* = 4)

PL growth factor content was analysed to identify potential mediators of senescence observed in long-term fibroblast cultures (Fig. 3a and b). The most abundant growth factors were the α-granule factors, including TGF-β1, platelet-derived growth factor 1 (PDGF) and insulin-like growth factor 1 (IGF-1), which largely agreed with previous reports (Rauch et al. 2011; Shanskii et al. 2013). However, PL did not induce IL-6 and IL-8 upregulation in fibroblasts (Fig. 3d–f). Moreover, the expression of FGF-2 and keratinocyte growth factor (KGF) that support keratinocyte ex vivo growth (Russo et al. 2020) was measured by qPCR in PL-expanded fibroblasts (Fig. 3d). Similarly, we analysed ECM protein expression in PL-expanded fibroblasts (Fig. 3c). Even after a 3-week expansion, similar levels of FGF-2, KGF, ColIII and fibronectin were expressed in PL-expanded fibroblasts, when compared to FBS-expanded fibroblasts. However, PL induced ColI expression in fibroblasts, compared to FBS, after a 3-week expansion period (*p* < 0.0001).

### Long-term expansion of fibroblasts in PL promotes survival of CD90^+^FAD^+^ subpopulation

**Fig. 4:**
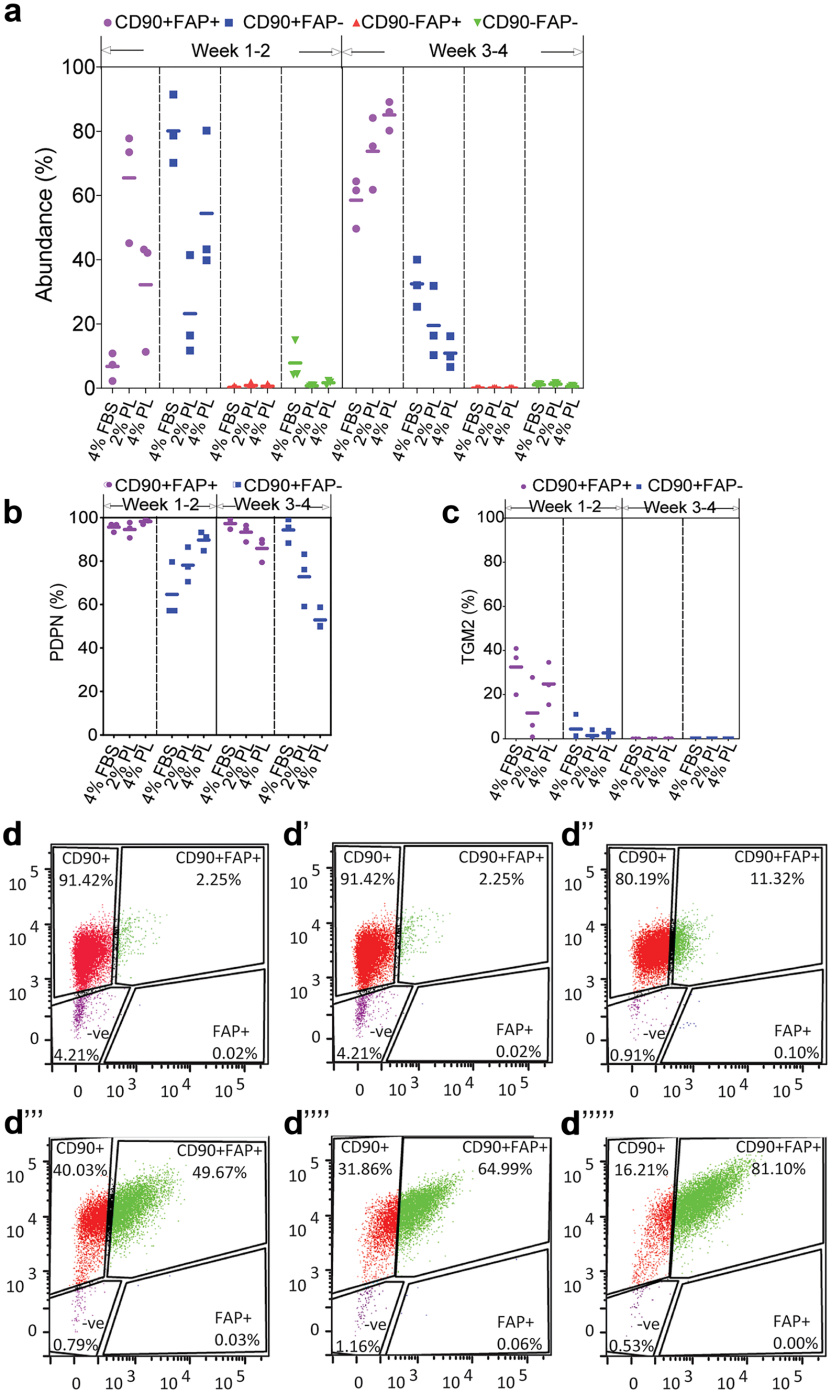
Resolution of fibroblast subpopulations expanded in PL-supplemented media. Freshly isolated dermal fibroblasts were expanded in 2% PL-, 4% PL- or 4% FBS-supplemented media as a control over 4 weeks. **a** The abundance of CD90 and FAP fibroblast markers were analysed by flow cytometry. The abundance of **b** PDPN and **c** TGM2 in CD90/FAP subpopulations were measured (*n* = 3 per group). **d** The gating strategy to separate CD90 and FAP subpopulations. Panels **d**–**d’’** represent fibroblasts in 4% FBS, 2% PL and 4% PL after 1–2 weeks of expansion, respectively. Panels **d’’’**–**d’’’’’** represent fibroblasts in 4% FBS, 2% PL and 4% PL after 3–4 weeks of expansion, respectively

Fluorescence-activated cell sorting (FACS) was employed to monitor phenotypic changes in PL-expanded fibroblasts. Cells were resolved into four subpopulations based on their CD90 and FAP surface marker expressions (Fig. 4d–d””’). The proportion of CD90^+^FAP^−^ (reticular phenotype), CD90^+^FAP^+^ (intermediate phenotype) and CD90^−^FAP^+^ (papillary phenotype) fibroblasts were determined after 1–2 weeks and 3–4 weeks of growth in PL vs FBS. Figure 4a shows a small proportion of CD90^+^FAP^+^ fibroblasts (6.8% ± 0.1%) after 1–2 weeks in FBS. This subpopulation increased to 65.5% (± 0.2%) after the fibroblasts were cultured for 1–2 weeks in PL and remained high (73.8% ± 0.1%) when cultured in PL up to 3–4 weeks. Conversely, the majority of fibroblasts in FBS for 1–2 weeks were CD90^+^FAP^−^ (80.1% ± 0.1%), but their abundance dropped to 32.5% (± 0.1%) after 3–4 weeks in culture. A similar decrease in the CD90^+^FAP^−^ subpopulation (23.2% ± 0.2%) was observed when FBS was substituted with PL for 1–2 weeks and remained low (19.5 ± 0.1%) after 3–4 weeks. CD90^−^FAP^+^ papillary fibroblasts accounted for < 1% of the total population in FBS and PL cultures. Therefore, they were not analysed further due to low abundance.

In order to further characterise the PL-expanded fibroblasts, the expression of podoplanin (PDPN) and transglutaminase (TGM2) was determined in CD90^+^FAP^+^ and CD90^+^FAP^−^ subpopulations (Fig. 4b and c). PDPN, a mucin-type transmembrane protein expressed in dermal fibroblasts, was expressed in the majority of CD90^+^FAP^+^ (intermediate phenotype) fibroblasts (95.7% ± 0.1%) after 1–2 weeks expansion in FBS. Its expression remained high in CD90^+^FAP^+^ subpopulation, when fibroblasts were expanded in 2% PL (94.6% ± 0.1%) and 4% PL (98.3% ± 0.1%) for 1–2 weeks. There was lower PDPN in CD90^+^FAP^−^ reticular fibroblasts expanded in FBS (64.7% ± 0.1%). However, after 1–2 weeks in 2% PL and 4% PL, CD90^+^FAP^−^ fibroblasts showed an increase in PDPN to 78.1% (± 0.1%) and 89.7% (0.1%), respectively. Longer 3–4-week expansion of CD90^+^FAP^−^ reticular fibroblasts in 2% PL did not change PDPN level (72.8% ± 0.1%), whereas 4% PL reduced PDPN levels to 52.9% (± 0.1%).

Unlike PDPN, TGM2 expression was not sustained in culture after 1–2 weeks, regardless of the media supplement. TGM2 decreased from 32.5% after 1–2 weeks to 0.1% after 3–4 weeks, in the CD90^+^FAP^+^ subpopulation, when expanded in FBS. A similar TGM2 decrease was observed in 3–4 weeks of PL-expanded cultures of this subpopulation. The majority of CD90^+^FAP^−^ reticular fibroblasts were TGM2 negative. Overall, data indicates a mixed early time point–surface marker expression in fibroblast groups. A longer culture period led to a more homogenous population. The FBS to PL transition shifted the fibroblast phenotype over time, as cells acquired a more intermediate and papillary phenotype at the expense of reticular features.

### Serum-free 3D-engineered skin structure

As a first step in assembling serum-free engineered skin, PL-expanded fibroblasts were seeded either on top or within a platelet-derived hydrogel, and their proliferation was analysed by cell counting and an Alamar Blue assay (Supplementary Data 1). The hydrogels were prepared either 2 h or 24 h prior to seeding at 100%, 25% and 10% concentration. Overall, fibroblasts grew faster when they were seeded on top of the hydrogel, rather than within the hydrogel (*p* = 0.0059). The gelation time did not have a significant effect on fibroblast expansion, although when the hydrogel was stiffer (set for 24 h), there was a trend towards better support of fibroblast attachment and growth.

**Fig. 5:**
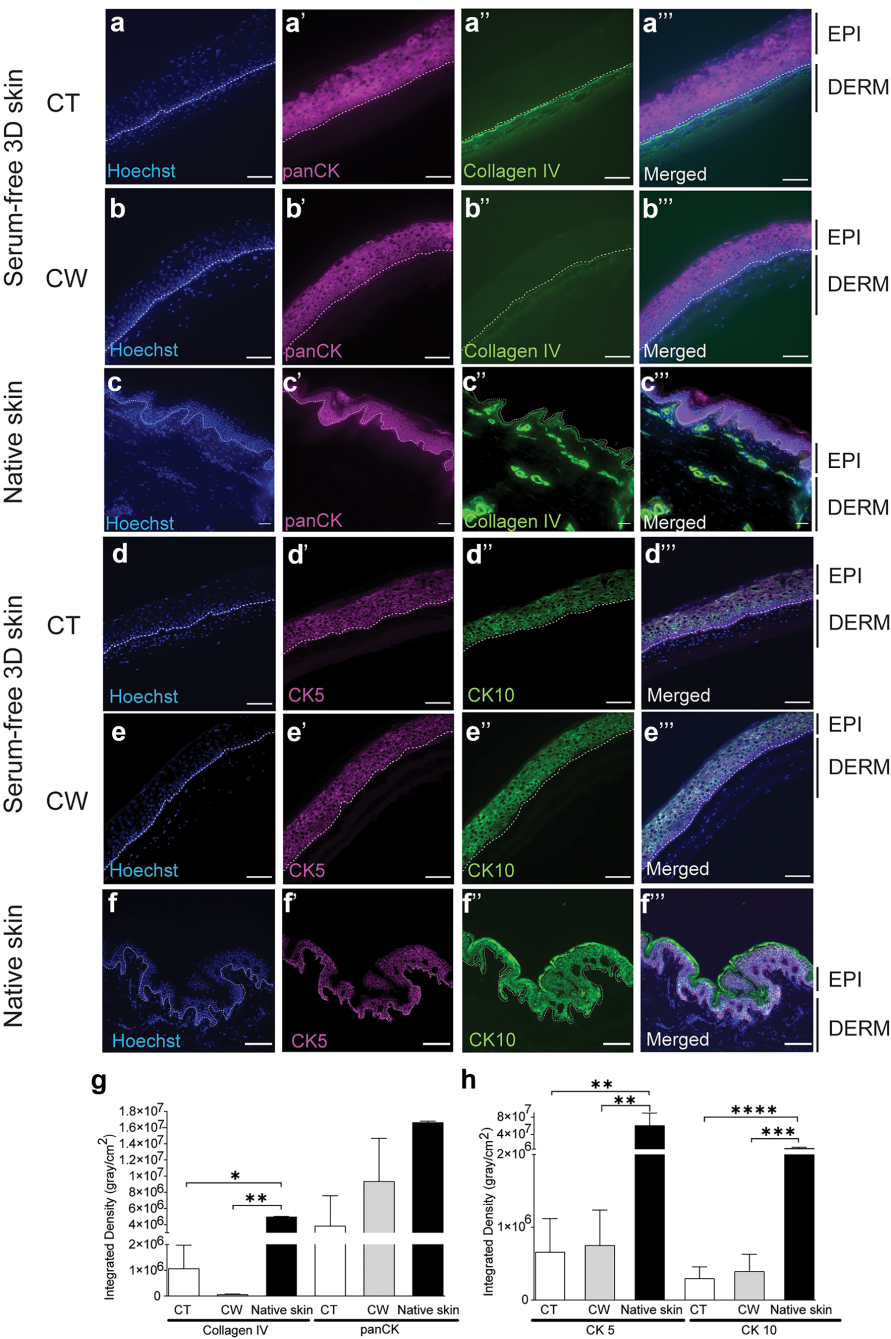
Serum-free 3D-engineered skin structure. **a**–**c** Collagen IV (ColIV, a basement membrane marker) and pan-cytokeratin (panCK) immunofluorescence staining showed higher ColIV deposition in the basement membrane when fibroblasts were seeded on top (CT = cells on top) of the hydrogel. The amount of cytokeratin in the epidermis was similar whether the fibroblasts were seeded on top or within the hydrogel (CW = cells within). **d**–**f** CK5 (a marker for interfollicular stem and progenitor keratinocytes) and CK10 (a differentiation marker) immunofluorescence staining confirmed CK5 expression in basal keratinocytes, when fibroblasts were seeded on top or within the hydrogel, although at a lower level, when compared to native skin. Basal keratinocytes did not express CK10, similar to native skin, regardless of whether fibroblasts were seeded on top or within the hydrogel. EPI, epidermis; DERM, dermis; and white dotted line, dermal/epidermal junction (scale bar 100 µm). **e** Integrated density quantitation for ColIV and panCK measured using FIJI software (*n* = 3). **f** Integrated density quantitation for CK5 and CK10 (* = *p* ≤ 0.05, ** = *p* ≤ 0.01, *** = *p* ≤ 0.001) (*n* = 4)

The 3D-engineered skin was assembled by seeding keratinocytes from the same individual on top of fibroblast-populated hydrogels and grown in a maturation media supplemented with 0.1% PL (Supplementary Data 2). Cytokeratin pan-expression showed that fibroblasts seeded on top or within the hydrogel-supported epidermal stratification (Fig. 5). There was a trend in higher basement membrane deposition (shown by ColIV expression) when fibroblasts were seeded on top of the hydrogel, but it did not reach significance (Fig. 5g). Conversely, there was a trend of reduced accumulation of differentiation marker, Keratin10, in neo-epidermis, if fibroblasts were seeded on top of the hydrogel and, therefore, in close proximity to keratinocytes (Fig. 5h).

**Fig. 6:**
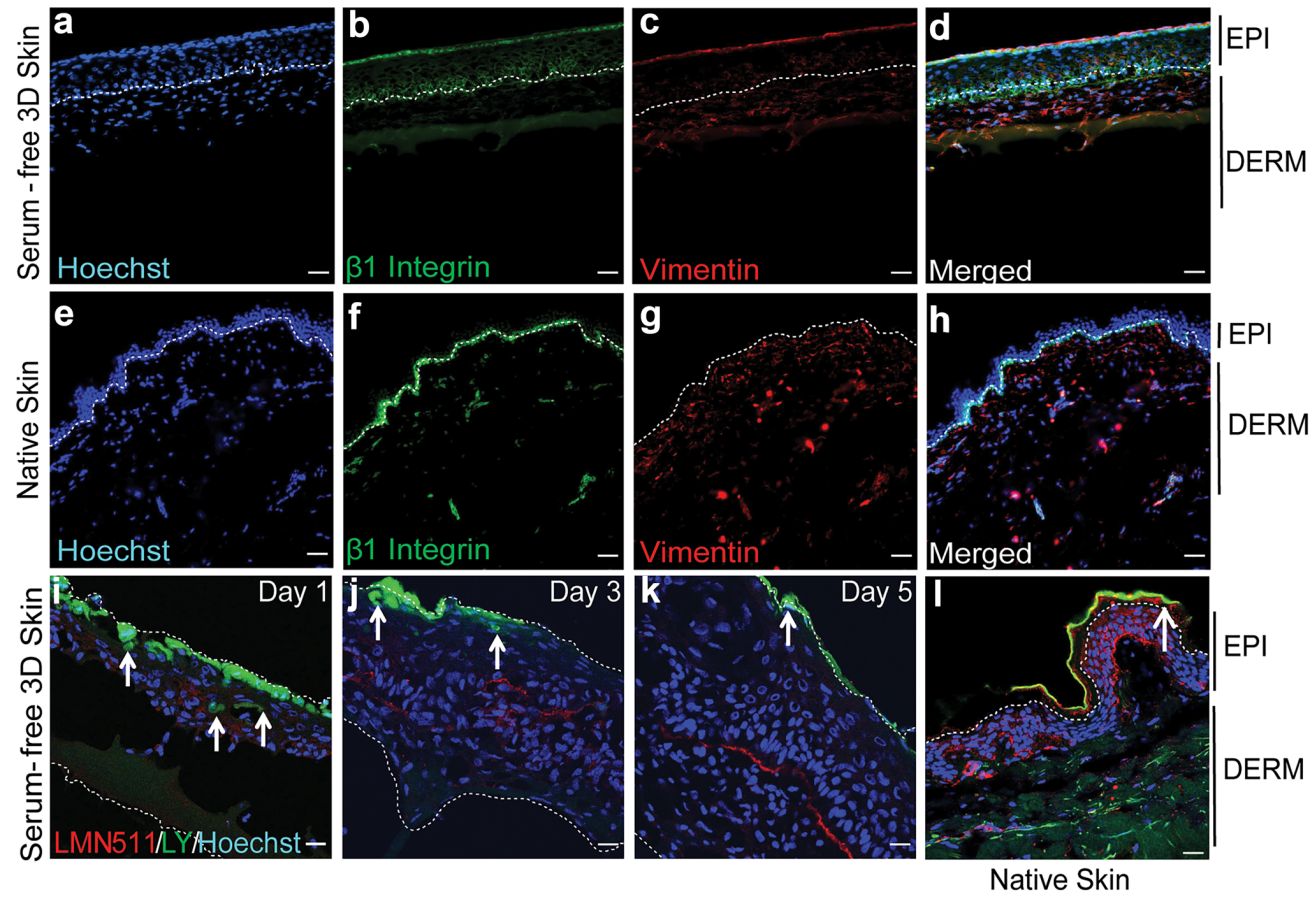
Functional analysis of serum-free 3D-engineered skin. **a**–**d** Serum-free 3D skin and **e**–**h** native skin sections were co-stained for CD29 (a marker for interfollicular stem and progenitor keratinocytes) and Vimentin (a fibroblast marker) on day-5 post keratinocyte seeding (*n* = 3) (scale bar 100 µm). EPI, epidermis; DERM, dermis; and white dotted line, dermal/epidermal junction. **i**–**l** The barrier function was detected by confocal microscopy showing reduced lucifer yellow (LY) penetration overtime on days 1, 3 and 5 post keratinocytes seeding, and it was compared to lack of LY penetration on native skin. Laminin-511 (LMN-511), in red, marks the basement membrane that divides dermal and epidermal compartments. LY is shown in green and the white arrows show the depth of cells from the surface that have taken up the LY dye. The figure is a representative of two independent experiments (scale bar 100 µm)

**Fig. 7:**
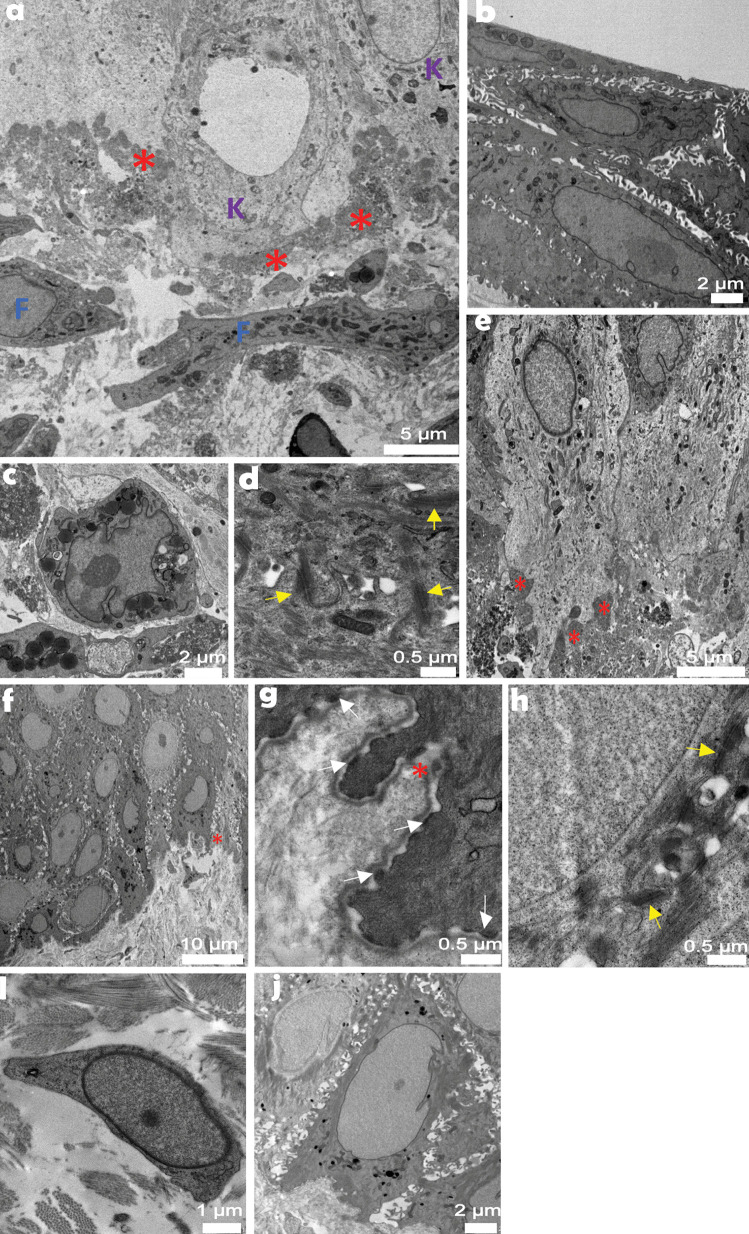
Transmission electron microscopy analysis of serum-free 3D-engineered skin. Atop basement membrane zone (**a**), tightly packed basal keratinocytes (**e**) formed a stratum, distinct to loosely spread fibroblasts below. Corneocytes (**b**), basal keratinocytes (**a**, **e**) and organelle-rich fibroblasts (**c**) observed across skin construct cross-section were complemented by desmosomes (**d**) and basal membrane ultrastructures. In between, a still disorganised basement membrane built up as a thick and non-linear entity. Native human skin provided an architecturally robust tissue organisation in the basement membrane zone (**f**). Fibroblast cytoplasm (**i**) was less organelle-packed. Basement membrane (**g**) was a thin, linear structure, desmosomes (**h**) linked neighbouring keratinocytes (**j**), while hemidesmosomes (**g**) adhered basal keratinocytes to the basement membrane. Red asterisk, basement membrane; K, keratinocyte; F, fibroblast; yellow arrow, desmosome; and white arrow, hemidesmosome. Scale bar values are in micrometres

CD29 and CK5 confirmed the persistence of stem and progenitor adult keratinocytes in serum-free 3D-engineered skin (Fig. 6a–d). There was also a gradual skin maturation, which resulted in the build-up of a barrier on the serum-free 3D-engineered skin, shown by reduction of lucifer yellow penetration into skin over time (Fig. 6i–k). The ultrastructure of the mature serum-free skin was analysed by TEM (Fig. 7a–e). Similar to native skin (Fig. 7f–j), the basal keratinocytes had finger-like microvillae (Norlen 2008). On the superficial layers of the skin, the differentiated keratinocytes were flattened, gradually losing their chromatin. The basement membrane was less rigid in engineered skin than it was in native skin. Consequently, basal keratinocytes lacked detectable hemidesmosomes which may reflect relatively free movement between dermis and epidermis through the hydrogel, compared to the native collagen-based dermis. Fibroblasts are seemingly more active than their counterparts in native skin containing a large number of vesicles, Golgi bodies and lipid droplets, typically observed in stimulated fibroblasts in culture (Gorin et al. 1982). Similar to native skin, some fibroblasts in engineered skin lie horizontally underneath the basement membrane maximising their surface towards basal keratinocytes. Desmosomes (attached to keratin intermediate filaments) were abundant between keratinocytes in engineered skin. SEM analysis (Supplementary Data 3) showed the porous (area 50.84 ± 0.17 µm^2^) neo-dermis covered by neo-epidermis (9.65 ± 0.5 µm thickness). The top view of the skin confirmed the presence of a mature epidermis, similar to native skin, with little detectable pores on the surface.

**Fig. 8:**
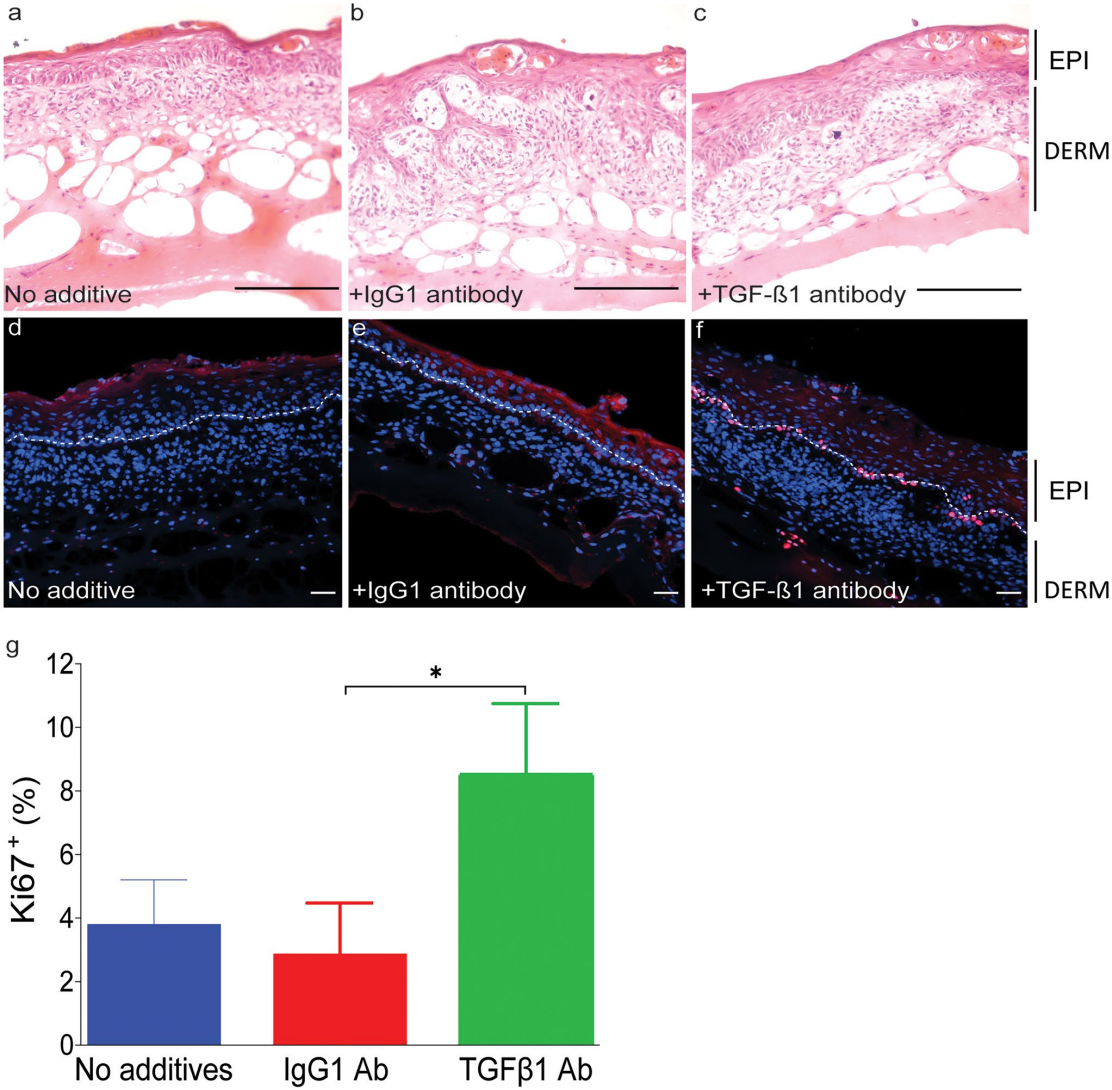
Serum-free 3D skin maturation is mediated, at least partly, through TGF-β1 signalling. Serum-free 3D-engineered skin was cultured in the presence of either TGFβ1 neutralising or IgG1 control antibodies. **a**–**c** The top panel shows haematoxylin and eosin staining on day 5 post keratinocyte seeding, in media alone, media plus isotype control antibody, and TGF-β1 neutralising antibody, respectively. Stratified epidermis was detectable even in the presence of TGF-β1 antibody. **d**–**f** Sections were analysed for the presence of Ki67 (a marker for proliferating cells) by immunofluorescence. EPI, epidermis; DERM, dermis; white dotted line, dermal/epidermal junction (scale bar 100 µm). **g** Ki67.^**+**^ (%) cells were counted in four fields of view per experiment for statistical analysis. There was significantly less proliferation in serum-free 3D skin in presence of TGF-β1 neutralising antibody compared to the isotype control antibody. Graph represents two independent experiments (* = *p* ≤ 0.05)

Skin maturation was mediated, at least partly, by TGF-β1 (Fig. 8). This was shown by incubating the serum-free 3D-engineered skin in presence of TGF-β1 neutralising antibody. The number of proliferative cells was identified by immunofluorescence using the Ki67 antibody. It was shown that proliferative cells were significantly reduced in presence of TGF-β1 neutralising antibody, compared to the non-specific antibody.

## Discussion

This study investigated the use of PL as a substitute for FBS in bioengineering 3D skin. A number of growth factors were identified in PL, some with reported opposing effects. In particular, PDGF, a growth factor present at physiological concentrations in PL, is a known stimulator of fibroblasts proliferation both in vitro and in vivo (Antoniades et al. 1991; Bonner et al. 1990), whereas TGF-β1 is believed to induce excessive ECM deposition in fibroblasts (Pakyari et al. 2013). It is the balance of growth factors in PL that drives its effect on tissue maturation. PL supported short-term fibroblast expansion in monolayer and keratinocyte maturation in 3D-engineered skin with a near-native architecture. However, long-term (7–8 weeks) expansion of fibroblasts in PL triggered senescence. Replication-induced senescence was analysed in two independent methods by measuring telomere length and cellular accumulation of β-galactosidase (El Backly et al. 2011).

Dermis can be divided into two distinct layers of papillary and reticular dermis. Fibroblasts isolated from each layer are genetically and phenotypically distinct. The two subpopulations have distinct but overlapping ECM and growth factor expression profiles. Reticular fibroblasts express higher levels of ECM, whereas papillary fibroblasts preferentially express growth factors that support epidermisation in engineered skin (Haydont et al. 2020). In this study, a pool of fibroblasts from full-thickness skin was isolated to include both papillary and reticular fibroblasts. We found that PL supplementation of fibroblasts did not change their KGF and FGF-2 expression at the RNA level, compared to FBS. However, ColI (and ColIII in 4% PL) was upregulated in PL-expanded fibroblasts. ColI is the main collagen found in dermis, and its deposition may have a positive effect on keratinocyte homeostasis in a co-culture.

The shift in the ECM expression profile of PL-expanded fibroblasts was likely due to the selective expansion of fibroblast subpopulations in PL. Dermal fibroblasts expanded in PL after 1–2 weeks, showed a reduction in the CD90^+^FAP^−^ subpopulation. Instead, the number of CD90^+^FAP^+^ fibroblasts increased. In dermis, CD90^+^FAP^−^ marks reticular fibroblasts, although their location is not restricted to the lower dermis. FAP marks the papillary fibroblasts, whereas fibroblasts that express both CD90 and FAP are found throughout the dermis, particularly in the intermediate region (Korosec et al. 2019). CD90 is a cell–matrix adhesion molecule and is activated in cultured cells. In vivo, CD90^+^FAP^−^ reticular fibroblasts are associated with fibrotic scaring, whereas CD90^−^FAP^+^ papillary fibroblasts are non-fibrotic (Chellini et al. 2018; Shook et al. 2018; Worthen et al. 2020). Here, we found that PL drives a shift in subpopulation’s expansion towards a less fibrotic phenotype in culture. The intermediate fibroblasts were expanded at the expense of reticular fibroblasts in PL-supplemented media which could have an additional benefit upon transplantation. The hierarchical analysis of the CD90^+^FAP^+^ subpopulation showed these cells are mostly PDPN^+^.

PL may activate the focal adhesion complex (that controls cell–matrix interaction) in keratinocytes in vitro. PL (20%) enhanced cell attachment to tissue culture flasks by activating focal adhesion in the HaCaT keratinocyte cell line, triggering ERK 1/2 activation independent from the NF-кB pathway (Ranzato et al. 2011). This was contradictory to another study showing NF-кB activation by (5%) PL in the NCTC 2544 keratinocyte cell line (El Backly et al. 2011). There are a number of possibilities causing differing results in the two studies. PL-activated pathways may in fact be cell line specific, or the response to PL may be concentration dependent. The current study presents the analysis of primary adult keratinocytes to overcome the cell line limitations. Here, we have shown that PL (in combination with a platelet-derived hydrogel) allows the formation of a serum-free 3D-engineered skin with the tissue architecture similar to native skin. This novel 3D skin can be used for replacing defective skin in patients or as an in vitro model to study the interplay between dermal-epidermal cell types.

Although PL at 0.5–4% did not increase the fibroblast proliferation rate, it upregulated ColI expression in this study. TGF-β1 is a known stimulator of ColI expression in fibroblasts during wound repair (Chong et al. 2020; Johnson et al. 2020). TGF-β1 also suppresses the expression of matrix metalloproteinases (MMPs), the enzymes that modulate the synthesis and degradation of the ECM (White et al. 2000). As a result, TGF-β1 has been implicated in aberrant scar formation pathologies such as keloids (Lee et al. 1999). The role of TGF-β1 during wound repair is rather complex. It has been shown that TGF-β1 accelerates wound repair in partial-thickness murine wounds through its stimulatory effect on fibroblasts. In full-thickness wounds, however, TGF-β1 delays wound closure by the inhibitory effect on keratinocyte migration (Tredget et al. 2005) and prolonging the inflammatory phase of wound healing through its intrinsic pro-inflammatory effect (Wang et al. 2006). In ex vivo, inhibition of the TGF-β1 signalling increased p63^+^ interfollicular stem cell proliferation and reduced differentiation capacity (Pinto et al. 2020). Therefore, it is likely that the TGF-β1 present in PL contributes to skin maturation and stratification in serum-free 3D-engineered skin. This was shown by reduced proliferation in presence of TGF-β1 neutralising antibody.

Few other commercially available PL products, such as Plus™ Cell Culture Supplement (Compass Biomedical) and Human Platelet Lysate (Stem Cell Technologies), already have or seek to obtain regulatory approval for in vitro expansion of therapeutic cells. PL prepared here from clinically expired platelet concentrates should be tested against other products for a performance comparison. Although PL is increasingly used for GMP-compliant expansion of cells as therapeutics, this study highlights the need for further characterisation of specific cell types expanded in PL to ensure the efficacy of the cell, and tissue therapy is maintained. It remains to be seen whether this combined cell and matrix therapy can improve wound repair in an animal model. We have previously shown that a platelet-derived hydrogel improves the vascularisation of full-thickness wounds in mice. The combined cell and matrix in a 3D-stratified skin structure in this study may not only be able to close wounds but also enhance vascularisation in difficult-to-heal wounds.

## Supplementary Information

Below is the link to the electronic supplementary material.

Supplementary file1 (TIF 290 KB)

Supplementary file2 (TIF 472 KB)

Supplementary file3 (TIF 467 KB)
