# Supplementary figures and images for: Platelet lysate can support the development of a 3D-engineered skin for clinical application
Source: Cell Tissue Res. 2022 Oct 22;391(1):173–88. doi: 10.1007/s00441-022-03698-7 (PMC9839813; doi:10.1007/s00441-022-03698-7)

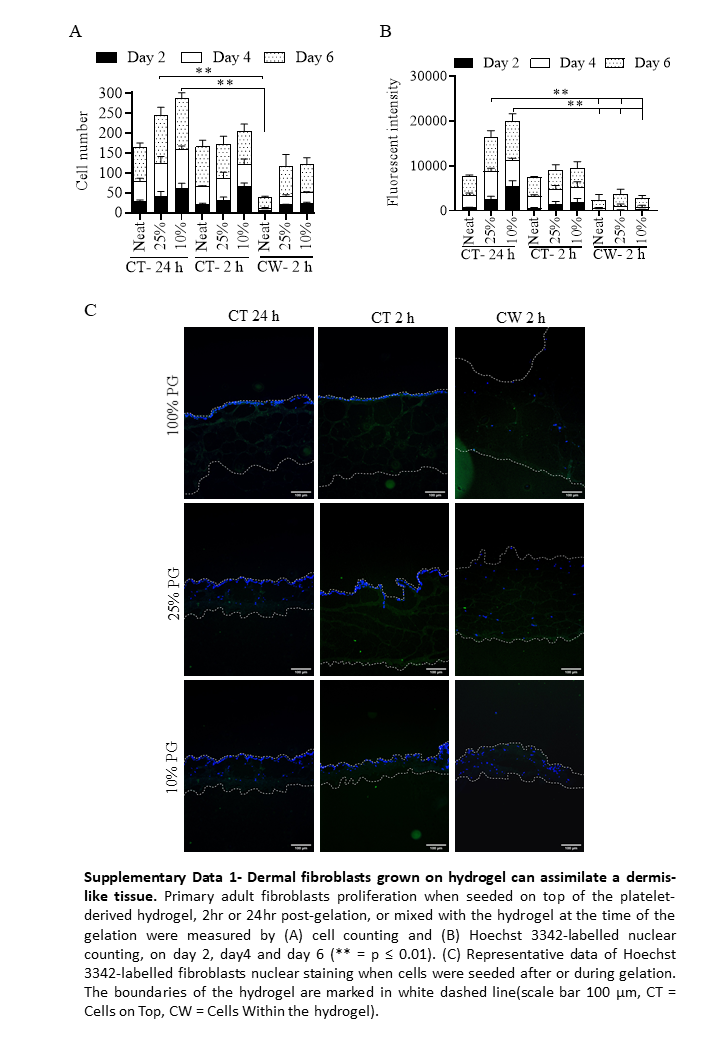

Supplement: Supplementary file 1 — Supplementary file1 (TIF 290 KB) [file 441_2022_3698_MOESM1_ESM.tif]

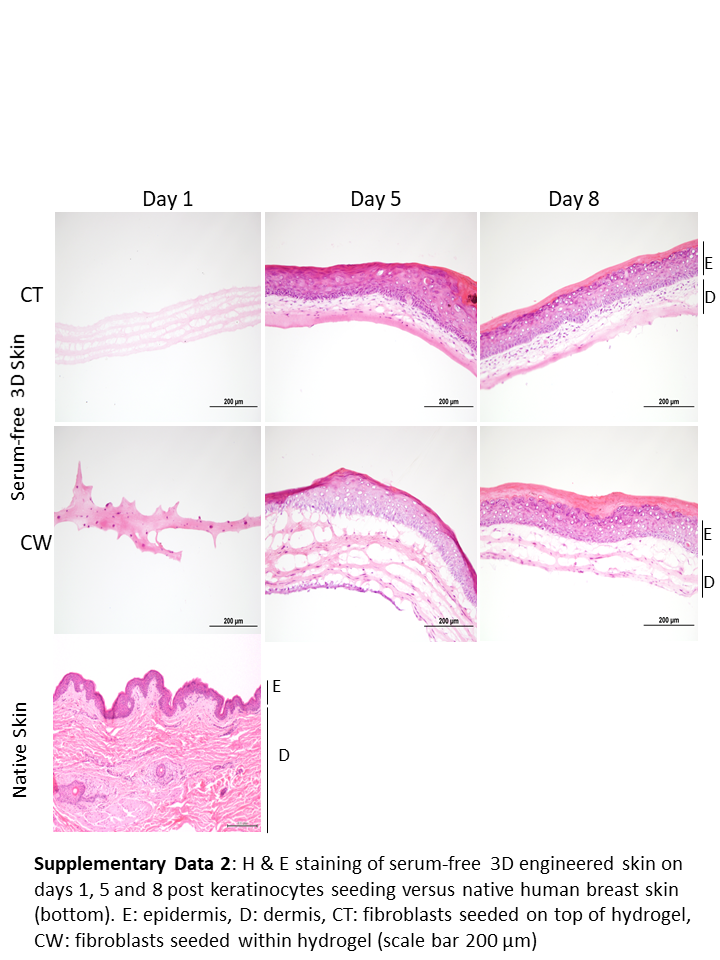

Supplement: Supplementary file 2 — Supplementary file2 (TIF 472 KB) [file 441_2022_3698_MOESM2_ESM.tif]

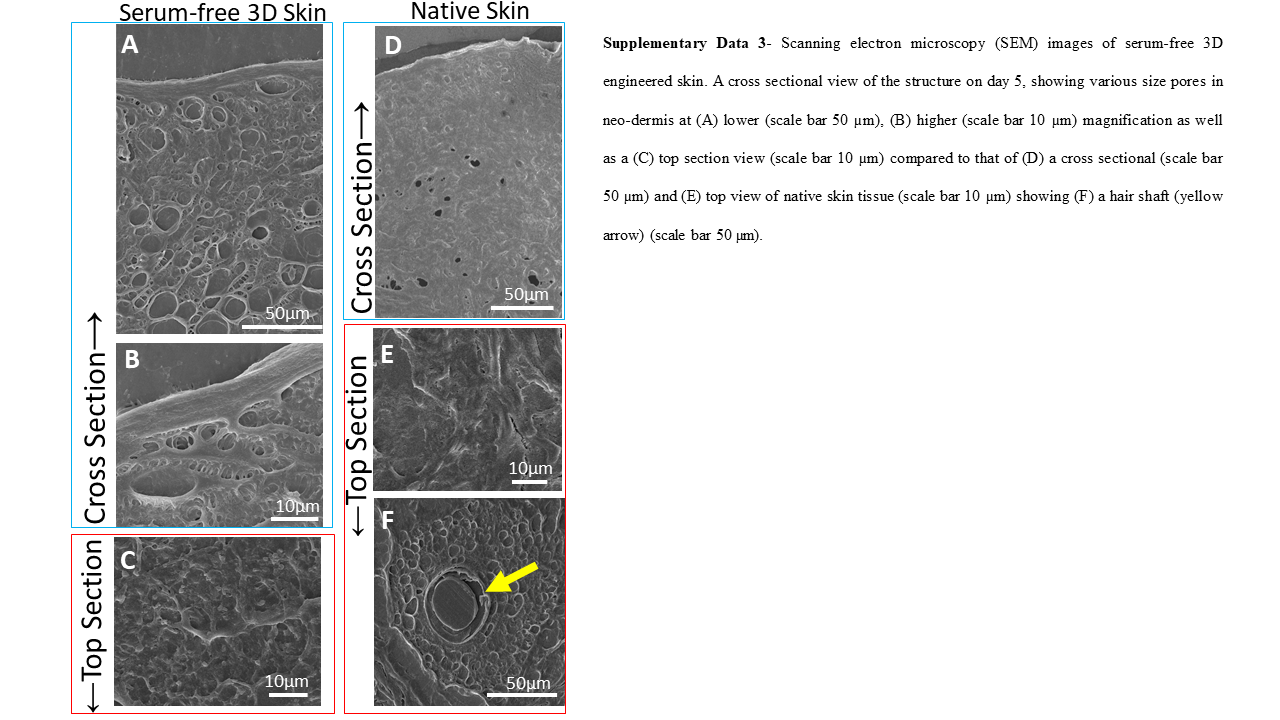

Supplement: Supplementary file 3 — Supplementary file3 (TIF 467 KB) [file 441_2022_3698_MOESM3_ESM.tif]
